# Effect of Radiation Emitted by Wireless Devices on Male Reproductive Hormones: A Systematic Review

**DOI:** 10.3389/fphys.2021.732420

**Published:** 2021-09-24

**Authors:** Sofwatul Mokhtarah Maluin, Khairul Osman, Farah Hanan Fathihah Jaffar, Siti Fatimah Ibrahim

**Affiliations:** ^1^Department of Physiology, Faculty of Medicine, Universiti Kebangsaan Malaysia (UKM), Kuala Lumpur, Malaysia; ^2^Department of Physiology, Faculty of Medicine and Health Sciences, Universiti Sains Islam Malaysia (USIM), Nilai, Malaysia; ^3^Centre of Diagnostic Science and Applied Health, Faculty of Health Sciences, Universiti Kebangsaan Malaysia (UKM), Bangi, Malaysia

**Keywords:** mobile phone, Wi-Fi, testosterone, follicle-stimulating hormone (FSH), luteinizing hormone (LH)

## Abstract

Exposure to radiofrequency electromagnetic radiation (RF-EMR) from various wireless devices has increased dramatically with the advancement of technology. One of the most vulnerable organs to the RF-EMR is the testes. This is due to the fact that testicular tissues are more susceptible to oxidative stress due to a high rate of cell division and mitochondrial oxygen consumption. As a result of extensive cell proliferation, replication errors occur, resulting in DNA fragmentation in the sperm. While high oxygen consumption increases the level of oxidative phosphorylation by-products (free radicals) in the mitochondria. Furthermore, due to its inability to effectively dissipate excess heat, testes are also susceptible to thermal effects from RF-EMR exposure. As a result, people are concerned about its impact on male reproductive function. The aim of this article was to conduct a review of literature on the effects of RF-EMR emitted by wireless devices on male reproductive hormones in experimental animals and humans. According to the findings of the studies, RF-EMR emitted by mobile phones and Wi-Fi devices can cause testosterone reduction. However, the effect on gonadotrophic hormones (follicle-stimulating hormone and luteinizing hormone) is inconclusive. These findings were influenced by several factors, which can influence energy absorption and the biological effect of RF-EMR. The effect of RF-EMR in the majority of animal and human studies appeared to be related to the duration of mobile phone use. Thus, limiting the use of wireless devices is recommended.

## Introduction

The use of mobile phones has become an essential part of our daily lives in the modern era. Mobile phone technology has rapidly evolved from a simple communication tool to a wireless device with multiple functions, such as internet browsing, gaming, video on demand, video conferencing, mobile TV, GPS navigation, and many more (Ezhilarasan and Dinakaran, 2017). Wi-Fi technology allows mobile phone users to access the internet via a Wi-Fi access point. Its versatility and widespread use expose people, including those in public places, to radiofrequency electromagnetic radiation (RF-EMR) (Miller et al., 2019).

The radiation emitted by Wi-Fi and all generations of mobile phones is classified as non-ionizing radiation, which falls within the microwave range (3–300 GHz) (Vishnu et al., 2011). First-generation (1G) and second-generation (2G) mobile phones operate at a frequency range of 850–1900 MHz (Vishnu et al., 2011), whereas third-generation (3G) mobile phones can reach up to 2,500 MHz (Ezhilarasan and Dinakaran, 2017). The most recent fourth-generation (4G) and fifth-generation (5G) mobile technologies operate at a wider and higher frequency range of 2–8 GHz and 3–300 GHz, respectively (Ezhilarasan and Dinakaran, 2017). However, unlicensed spectrum bands of 2.4 (802.11b/g/n/ax) and 5 GHz (802.11a/h/j/n/ac/ax) are used by Wi-Fi devices (Zhang et al., 2014).

The impact of RF-EMR on the human body is measured using a standardized unit known as specific absorption rate (SAR). SAR measures how much energy from radiofrequency (RF) waves is absorbed by human tissues and is calculated as watts per kilogram (W/kg) (Hochwald et al., 2014). According to the United States Federal Communications Commission (Federal Communications Commission, 1996), SAR limit should not exceed 1.6 W/kg as averaged over one gram of tissue. Additionally, the International Commission on Non-Ionizing Radiation Protection (ICNIRP, 2020) recommends a limit of 2 W/kg for head and trunk exposure over 10 grams of tissue. Based on the recommendation, each country and wireless device manufacturer produce devices which adhere to the SAR limits. In other words, it varies between country of origin but within range to avoid damaging effects to the human tissues.

The effect of these devices on human health, specifically on male reproduction, is frequently overlooked. Mobile phones and Wi-Fi devices have a detrimental impact on the male reproductive system, which is reflected in the sperm quality (Houston et al., 2016; Jaffar et al., 2019). The biological effects of RF-EMR emitted from wireless devices can be categorized as thermal and non-thermal (Behari, 2010). Thermal effects are associated with the heat created by holding mobile phones close to the body and conversing for extended periods of time (Behari, 2010). The non-thermal mechanism of RF-EMR is associated with the formation of reactive oxygen species (ROS) and the induction of oxidative stress (Saliev et al., 2018). Both heat and oxidative stress were linked to disruption of the germ cell cycle and lead to the increased of sperm cells apoptosis in the testis (Kesari et al., 2011). The loss of the germ cells will ultimately affect the hormonal balance as it is one of the important components in the hypothalamus-pituitary-gonadal (HPG) axis.

RF-EMR caused histological aberrations in the testes, testicular tissue atrophy, decreased testosterone levels, and a subsequent deterioration in sperm quality (Adah et al., 2018). Among the reproductive parameters studied, less attention has been paid to the effects of wireless devices on male reproductive hormones. The intricate interaction of hormones involved in the hypothalamic–pituitary–testes axis, particularly gonadotropin-releasing hormone (GnRH), follicle-stimulating hormone (FSH), luteinizing hormone (LH), testosterone, and estrogen, are essential for male reproductive functions. These hormones may be affected by RF-EMR exposure, which may result in male reproductive dysfunction and infertility. However, no conclusive evidence has proven that wireless devices are harmful to male reproductive hormones other than testosterone. As a result, this review focuses on the most recent scientific evidence of the effects of mobile phone and Wi-Fi exposure on the hypothalamus–pituitary axis and male reproductive hormones. This review also discusses the factors that influence the total energy absorption of RF-EMR emitted by wireless devices and their importance.

## Method

### Search Strategy

Academic journals were retrieved from MEDLINE and PUBMED databases from 1995 to 2020. Literature search was done using the following keywords: “WiFi OR Wi-Fi OR wireless fidelity OR WiFi router OR Wi-Fi router OR mobile phone OR cell phone OR electromagnetic radiation OR radiofrequency radiation” AND “estrogen OR estradiol OR testosterone OR androgen OR LH OR luteinizing hormone OR FSH OR follicle-stimulating hormone OR gonadotropin.” Additional journals were also identified through Google scholar using a similar set of keywords.

### Study Inclusion and Exclusion Criteria

The eligible articles included in this review were original articles that were full-length and written in English. The study must include a report on the effect of Wi-Fi or mobile phones on male reproductive hormones, namely, LH, FSH, testosterone, or estrogen, in animals or humans. Studies that used mobile phones with frequencies between 850 and 1,850 MHz and Wi-Fi devices that operate at 2.45 GHz were included in this review. Studies on RF radiation generated from a specialized chamber with these frequencies were also included. Studies that used radiation below the range of mobile phone frequency (850 MHz) and Wi-Fi operated at 5 GHz were excluded.

### Data Extraction and Management

The study selection involved two steps. First, the title and abstract of the articles were screened. Studies that did not meet the inclusion criteria were excluded. Subsequently, the full text of the selected articles was retrieved and filtered based on the same inclusion and exclusion criteria. Data extraction was done on the sample size, animal species, age of humans and animals involved, radiation frequency, exposure setting, SAR, and/or power density values. These data were then associated with the findings on male reproductive hormones, namely, LH and FSH, testosterone, or estrogen.

## Results

### Studies Selected

The initial database search identified 451 articles, of which 56 were from PubMed and 395 were from MEDLINE. Another three articles from Google Scholar were also included. Eighty-three articles were removed after finding duplicates, and 349 articles were further excluded after title and abstract review. Finally, another two articles were excluded after full-text review because both articles were retracted due to inadequate evidence to justify the accuracy of the data presented and the overlapping data and figures between these two articles. Nineteen eligible full-text articles were included in the systematic review. The PRISMA flow diagram of the literature search is presented in Supplementary Figure 1.

### Study Characteristics

#### Animal Study

Fourteen of the 19 eligible articles documented the effect of mobile phone application on male reproductive hormones. Most of the studies used rodents as their subjects, including Wistar albino rats (*n* = 7), Sprague Dawley rats (*n* = 4), Swiss albino mice (*n* = 1), and C57BL/6 mice (*n* = 1). One study had used pure bred New Zealand white rabbits (*n* = 1, Supplementary Table 1).

The study design, including the age of the animals, the frequency of radiation, the type of radiation generator used, the distance between cages and generator, and the duration of exposure, differs in several ways. The age of the animals used in the studies vary greatly. The animals were in different life stages, namely, prenatal age (Sehitoglu et al., 2015), weaning age (3-week-old mice) (Shahin et al., 2018), adolescence (4-week-old rats) (Ribeiro et al., 2007), early adulthood (8–10-week-old rats) (Meo et al., 2010; Kesari and Behari, 2012; Çetkin et al., 2017), and adulthood (12–20-week-old mice and rats) (Ozguner et al., 2004; Sepehrimanesh et al., 2013; Zang et al., 2016; Yahyazadeh et al., 2020).

Variations in RF-EMR frequency and the type of generator used also exist. In the studies that used mobile phones, the frequency applied includes 850–950 (Kesari and Behari, 2012; Zang et al., 2016; Çetkin et al., 2017) and 1,800–1,850 MHz (Ribeiro et al., 2007; Shahin et al., 2018). A study used a dual-band 900/1,800 mobile phone as RF-EMR source (Oyewopo et al., 2017). Other studies used a 900 MHz continuous wave electromagnetic generator (Ozguner et al., 2004; Sepehrimanesh et al., 2013; Sehitoglu et al., 2015; Yahyazadeh et al., 2020), a 900 MHz RF wave (Azimzadeh and Jelodar, 2019), and a 950 MHz simulated mobile phone (Oskouyi et al., 2014).

Another factor found to be inconsistent between studies is the distance between the study subject (animals) and generator. Most of the studies mentioned that the mobile phone or antenna was placed near the animals. In these studies, the mobile phone or antenna was placed centrally under (Sehitoglu et al., 2015; Zang et al., 2016; Çetkin et al., 2017), inside (Meo et al., 2010; Oskouyi et al., 2014), and above the cages (Ozguner et al., 2004; Kesari and Behari, 2012). Another study positioned the mobile phone 50 cm from the cages (Gevrek et al., 2017). The furthest distance applied was 1 m from the generator (Azimzadeh and Jelodar, 2019).

The duration of RF-EMR exposure also varied. The shortest exposure was 30 min/day for 20 days (Ozguner et al., 2004). The longest exposure duration was 24 h/day for 30 days (Zang et al., 2016). Nonetheless, most studies reported SAR values lower than the FCC and ICNIRP recommendations, with the exception of the studies by Yahyazadeh et al. (2020) and Saygin et al. (2016), which reported SAR values of 2 and 3.21 W/kg, respectively.

In animal studies, 85% (12 out of 14 studies) reported that RF-EMR from mobile phone causes a significant decrease in testosterone levels (Ozguner et al., 2004; Meo et al., 2010; Kesari and Behari, 2012; Sepehrimanesh et al., 2013; Oskouyi et al., 2014; Sehitoglu et al., 2015; Zang et al., 2016; Gevrek et al., 2017; Oyewopo et al., 2017; Shahin et al., 2018; Azimzadeh and Jelodar, 2019; Yahyazadeh et al., 2020). Furthermore, Meo et al. (2010) demonstrated a decreasing pattern in the serum testosterone level with increasing exposure duration. The subjects exposed to mobile phone for 60 min/day showed lower testosterone level compared with those with an exposure of 30 min/day (Meo et al., 2010). However, another two studies (15%) reported no significant changes in testosterone level (Ribeiro et al., 2007; Çetkin et al., 2017). Ribeiro et al. (2007) reported this finding after exposing adult Wistar rats to low-intensity pulsed RF radiation from a mobile phone for a short period. However, no study on the effect of mobile phone exposure on male estrogen levels was found.

Findings on the serum levels of gonadotropin hormones (LH and FSH) following mobile phone exposure are inconclusive. Two animal studies out of six (33%) reported a significant decrease in gonadotrophin levels. Oyewopo et al. (2017) reported a significant decrease in FSH and LH levels after exposure to mobile phones for 2–3 h/day. These findings are consistent with the study by Gevrek et al. (2017), which also reported a significant decrease in LH serum levels in the RF-EMR-exposed group. Other studies (two out of six studies, 33%) reported a significant increase in serum FSH and LH after mobile phone exposure (Sepehrimanesh et al., 2013; Zang et al., 2016). In comparison, two studies (33%) found no significant differences in FSH and LH levels in the exposed group (Ozguner et al., 2004; Çetkin et al., 2017). These variations in hormonal effects caused by RF-EMR exposure could be attributed to differences in study design, which influence energy absorption and biological effect.

The number of studies on the possible effects of 2.45 GHz Wi-Fi RF-EMR exposure on male reproductive hormones is very few, and the findings are inconsistent (Supplementary Table 2). Kumar et al. (2011) discovered a significant reduction in the testosterone level of adult male Wistar albino rats after long-term exposure (2 h/day for 60 days) to 2.45 GHz Wi-Fi with a SAR of 0.014 W/kg. By contrast, Saygin et al. (2016) reported no considerable decrease in testosterone levels in the RF-EMR-exposed group using a higher SAR value of 3.21 W/kg for 3 h/day for 30 days. Differences in SAR values, exposure duration, and exposure systems used in the studies could explain the contradictory findings. Kumar et al. (2011) had lined the exposure cage with radar-absorbing material and then positioned the cage vertically to irradiate all of the animals uniformly at a single power level. In comparison, Saygin et al. (2016) used a rotating carousel with 12 rats in a cylindrical restraint at the same time. This specialized laboratory equipment was developed to ensure the consistency of RF-EMR exposure.

#### Human Study

The effect of RF-EMR emitted from mobile phones on male reproductive hormones in human subjects was reported in three articles (Supplementary Table 3). Two articles were prospective cohort studies (Djeridane et al., 2008; Eskander et al., 2012), and one was a retrospective cohort study (Gutschi et al., 2011). The duration of the prospective cohort studies varied greatly from 4 weeks (Djeridane et al., 2008) to 6 years (Eskander et al., 2012). Except for one study which include older age up to 60 years old (Eskander et al., 2012), all the studies included men in their reproductive age. Djeridane et al. (2008) and Eskander et al. (2012) excluded subjects with a history of smoking and alcohol consumption, systemic diseases, orchitis, and varicocele. Djeridane et al. (2008) specified stressful conditions (work, unusual sleep pattern), high-intensity electromagnetic field (EMF) exposure, mobile phone use before the study, neuropsychiatric disease, and recent transcontinental flight as their exclusion criteria.

The serum testosterone level after RF-EMR exposure from a mobile phone was reported differently in all three studies. In a long-term 6-year survey that involved volunteers, testosterone levels showed a gradual decrease with increasing exposure time; the most substantial decrease occurred after 6 years of exposure (Eskander et al., 2012). Another study with a short duration of mobile phone exposure (2 h/day for 5 days in 4 weeks) found no substantial changes in testosterone levels (Djeridane et al., 2008). A retrospective study of men attending infertility clinics found that mobile phone users have remarkably high testosterone levels (Gutschi et al., 2011). In the same study, the exposed group had a considerable decrease in LH levels but no remarkable differences in FSH levels. However, the duration of mobile phone exposure was not mentioned in this study. Nevertheless, no studies on the effect of Wi-Fi exposure on the male reproductive hormones of humans were found.

## Discussion

In modern society, exposure to the RF-EMR emitted by various wireless devices is unavoidable. The amount of RF-EMR exposure also remarkably increases as we live close to a large number of actively transmitting Wi-Fi devices. Thus, its potentially harmful effects on humans are raising public concern. This review will discuss the impact of RF-EMR emitted from wireless devices on male reproductive hormones.

### Effect of Mobile Phone and Wi-Fi on the Male Reproductive Hormones of Animals

#### The Impact of RF-EMR on Gonadotrophin

The effect of mobile phone exposure on gonadotrophic hormones varied depending on the intensity of the exposure across studies. An exposure to 900 MHz RF-EMR increased serum FSH, LH, and prolactin levels in male Sprague-Dawley rats (Sepehrimanesh et al., 2013). Prolonged exposure to 900 MHz mobile phone for 24 h/day also elicits the same elevation in LH level in mice (Zang et al., 2016). Sepehrimanesh et al. (2013) also discovered an increase in activin b and a decrease in inhibin b, which indicate that the pituitary gland's feedback mechanism was unaffected and thus results in an increase in FSH level. Furthermore, an increase in prolactin causes an increase in LH levels. However, RF-EMR damage to Leydig cells causes an insufficient response to LH, which results in a lower testosterone level. The author hypothesized that RF-EMR exposure from a mobile phone could impair testicular function without affecting pituitary function. This claimed was based on the LH elevation and reduced testosterone level (Zang et al., 2016).

By contrast, higher-intensity RF-EMR exposure results in a decrease in serum LH and FSH (Gevrek et al., 2017; Oyewopo et al., 2017). This finding implied that mobile phones may harm the hypothalamus or pituitary. In a study conducted by Oyewopo et al. (2017), young adult Wistar rats were exposed to dual-band EGSM 900/1,800 MHz mobile phones for 1–3 h/day for 28 consecutive days. Gevrek et al. (2017) used two mobile phones (Nokia 3310 and NHM-5NX) in talking mode on Wistar albino rats for 4 h/day for 6 weeks and found a decrease in LH, FSH, and testosterone; thus, the author postulated that mobile phones might directly affect the hypothalamus or pituitary gland. Differences in the animal species and size, as well as radiation intensity, can affect total energy absorption by the animal body and, as the biological effect of RF-EMR. A brief exposure (30 min/day for 20 days) has no remarkable effect on LH and FSH levels (Ozguner et al., 2004). Thus, the effect of mobile phone exposure on gonadotrophic hormones remains elusive and varied depending on the level of exposure.

Minimal evidence supports the effects of RF-EMR on the pituitary gland and gonadotrophic hormones. The anterior pituitary gland secretes LH and FSH in response to GnRH from the hypothalamus. FSH causes Sertoli cells to produce androgen-binding protein, inhibin, and activin (Cooke et al., 2017), whereas LH stimulates the secretion of testosterone by testicular Leydig cells (Cooke et al., 2017). These hormonal regulations by the hypothalamus and anterior pituitary are essential for male reproductive functions. RF-EMR emitted from mobile phones can cause thermal effects as manifested by the elevation of temperature and EMF strength value on the hypothalamus and pituitary gland after mobile phone exposure (Uluaydin et al., 2020). The penetration of RF-EMR on the hypothalamus and pituitary gland is deeper in lower frequency bands (700 and 900 MHz) (Uluaydin et al., 2020). This theory is further supported by the HPA axis's deregulation after chronic mobile phone exposure (Singh et al., 2020). Nonetheless, the RF-EMR's effect and mechanism on the hypothalamus–pituitary–testes axis is still questionable and necessitates additional research. Supplementary Figure 2 depicts potential mechanisms for RF-EMR exposure's effect on gonadotrophin and testosterone secretion.

#### The Impact of RF-EMR on Testosterone

The reduction in testosterone in most animal studies demonstrated that Leydig cells are vulnerable to the RF-EMR emitted by mobile phones and Wi-Fi devices. The reduction in Leydig cells through injury in cell structure and function together with a reduction in testosterone were found after mobile phone exposure on rodents (Wang et al., 2003; Yahyazadeh et al., 2020). Moreover, the RF-EMR emitted by mobile phones and Wi-Fi cause increased oxidative stress (Kumar et al., 2011; Oyewopo et al., 2017) and apoptosis in Leydig cells (Sehitoglu et al., 2015). These RF-EMR-induced changes in Leydig cell histology and impairment could be explained by oxidative stress and direct RF-EMR effects, which cause changes in protein kinase C in Leydig cells (Wang et al., 2003).

In any of the studies that have been conducted, there is no evidence that the loss of germ cells affects male reproductive hormones. Only one study included germ cells as a study parameter. Yahyazadeh et al. (2020) discovered that the total number of primary spermatocytes and spermatids was significantly lower in the exposed group. They did not, however, correlate this data with hormonal levels.

#### Factors Influencing RF-EMR's Biological Effect

The total energy absorption of RF-EMR by the tissue is affected by various factors, such as biological complexity (species, strain, age, and sex) and engineering complexity (RF dosimetry, modulation, exposure parameters, and time-intensity factor) (Chou et al., 1996; Panagopoulos et al., 2013). The intensity of the RF field and exposure duration are crucial parameters that can cause a biological effect (36). Changes in reproductive hormones were observed in long-term exposure groups (Meo et al., 2010; Sepehrimanesh et al., 2013; Zang et al., 2016; Oyewopo et al., 2017). Thus, the severity of the effect of RF-EMR is duration dependent. A low (8 h/day) and middle (16 h/day) exposure to 900 MHz mobile phone for 30 days in mice did not affect the testosterone synthesis function of the testis, but high (24 h/day) exposure showed a reduction in testosterone level (Zang et al., 2016). Besides, we also discovered that three out of four studies (75%), which found no significant changes in reproductive hormones, were due to low intensity and short duration of exposure to mobile phone or Wi-Fi devices. In a study conducted by Ribeiro et al. (2007), a short duration of mobile phone exposure (1 h/day) showed no remarkable testosterone level changes. The author concluded that short-term exposure to the RF-EMR emitted by mobile phones does not cause impairment in testicular function in rats (Ribeiro et al., 2007).

Another factor that contributes to the biological effect of RF-EMR is RF dosimetry. RF dosimetry is defined as the quantification of the magnitude and distribution of RF-EMR energy absorption in an object (Chou et al., 1996). According to the National Toxicology Program of the United States, the exposure system used in the studies must deliver a uniform dose of RF field (Capstick et al., 2017). This requirement can be accomplished by uniformly distributing field directions to test subjects. A specialized exposure chamber, such as a carousel, anechoic, or reverberation chamber, improves RF dosimetry uniformity and eliminates potential localized heating caused by mobile phone-induced RF-EMR (Gong et al., 2017). A study conducted by Kesari and Behari (2012) shows an example of this phenomenon. The RF-exposed group of rats were placed in a Plexiglas cage with anechoic material, and their mobility was restricted during the exposure period. The variation in power density within each animal cage was discovered to be minimal (Kesari and Behari, 2012). However, in some animal studies, a mobile phone was placed in the cage as an RF-EMR source. As a result, the individual animal is subjected to an ill-defined exposure. This setup also fails to meet current RF dosimetry requirements for proper experimental data analysis (Kuster et al., 2006).

SAR, one of the most used dosimetry quantities, is defined as the rate of energy absorption per unit mass (Chou et al., 1996). The size, geometry, position, dielectric permittivity, and electric conductivity of the exposed object (animal), as well as the zone of exposure (near- or far-field), can have an impact on SAR value (Stuchly et al., 1985; Wu et al., 2010). Wu et al. (2010) studied the whole-body SAR in newborn and young rats exposed to 2.45 GHz RF-EMR. During the experiment, pregnant rats and newborn rats were free to move around in a reverberation chamber. They discovered using finite difference time domain that the whole-body SAR decreases after the fourth postnatal day because of the rats' body size and weight growth (Wu et al., 2010). Similarly, Peyman (2011) discovered that dielectric properties and SAR decrease with age. Changes in the water content and organic composition of tissues cause these changes in dielectric properties with age. The findings shed some light on potential differences in the assessment of exposure for children and adults.

Furthermore, a variation in whole-body SAR of up to 35% is caused by animal behavior or relative position for multiple rats (Wu et al., 2010). A mouse exposed to 2.45 GHz showed the highest whole-body average SAR in parallel orientation compared with facing orientation and standing up straight position (Wang et al., 2002). The electric field was parallel to the mouse in parallel orientation but perpendicular to the mouse in the other two positions. Besides, near- and far-field radiation can have an impact on the SAR. Generally, a near-field is one wavelength (λ) or less away from the source of radiation, whereas a far-field is about two wavelengths (2λ) away from the antenna (Occupational Safety Health Administration, 1990). Toivonen et al. (2019) demonstrated that the ICNIRP limit for localized exposure (2 W/kg) could be reached if the distance between the antenna and the body is <24 cm. As a consequence, when people hold their phones next to their heads or wear them on their bodies, they may be exposed to very intense near-field radiation from the device at close or touching distances.

In summary, a detailed study design, including animal selection, dosimetry analysis, and exposure assessment, are required for *in vivo* studies on the RF-EMR exposure of rodents to enable the correct interpretation of biological outcomes.

### Effect of Mobile Phone and Wi-Fi on the Male Reproductive Hormones of Humans

SAR is distributed in a non-uniform way in the human body and is typically highest in the body part closest to the device (Faruque et al., 2011). For example, when men carry their phones in their side pockets, which are very close to their testicles, they expose their testicles to harmful RF-EMR (Okechukwu, 2018). Mobile phones placed less than 15 cm from the testes have a harmful SAR value on the testes (Bhat, 2017). As a result, the testicular function, including its endocrine function, may be affected.

In human studies, the biological effect can be seen in higher-intensity RF-EMR emitted from a mobile phone over a longer period of exposure. The long-term use of 950 MHz mobile phones (6 years) can reduce testosterone levels (Eskander et al., 2012). The testosterone reduction seen in the studies was time dependent with a gradual decrease over a longer period of exposure. RF-EMR also has a negative effect on the anterior pituitary gland as evidenced by a decrease in adrenocorticotrophic hormone (ACTH), cortisol levels, and thyroid hormones (Eskander et al., 2012). Thus, RF-EMR appears to have a negative relationship with the anterior pituitary gland and its hormones.

This theory is supported by a retrospective study, which reported a decrease in LH level in mobile phone users (Gutschi et al., 2011). The study, on the other hand, discovered an increase in testosterone levels in the same group. They postulated that RF-EMR from mobile phone could cause Leydig cell hyperplasia, which results in increased testosterone levels but impaired reproductive functions (Gutschi et al., 2011). Decreased sperm quality validates the impairment of men's reproductive functions caused by mobile phones (Gutschi et al., 2011). In addition, a short duration of mobile phone exposure (2 h/day, 5 days/week for 4 weeks) revealed no effect on reproductive hormones (Djeridane et al., 2008). Nonetheless, because there are only three articles available, it is impossible to conclude the effect of RF-EMR on human male reproductive hormones. Besides, these studies also overlooked the general decline in testosterone with age. Thus, more research is needed to confirm the effect of RF-EMR on male reproductive hormones, including the hypothesis of Leydig cell hyperplasia.

## Conclusion

Existing animal and human data on the effect of RF-EMR emitted from wireless devices on male reproductive hormones are inconsistent and difficult to evaluate due to the heterogeneity of study design. However, most studies are consistent with the assertion that long-term exposure to RF-EMR from mobile phones and Wi-Fi devices can disrupt male reproductive hormones, particularly testosterone. Thus, avoiding long-term and excessive use of mobile phone is advisable to reduce the detrimental effect of RF-EMR.

## Data Availability Statement

The original contributions presented in the study are included in the article/Supplementary Material, further inquiries can be directed to the corresponding author.

## Author Contributions

All authors have read and agreed to the published version of the manuscript. SM: writing original draft preparation. SM, SI, KO, and FJ: writing—review and editing. SI, KO, and FJ: supervision. KO: funding acquisition. All authors contributed to the article and approved the submitted version.

## Funding

This work was supported by Universiti Kebangsaan Malaysia (Research grant: FRGS/1/2019/SKK06/UKM/02/3 and FF-2021-200).

## Conflict of Interest

The authors declare that the research was conducted in the absence of any commercial or financial relationships that could be construed as a potential conflict of interest.

## Publisher's Note

All claims expressed in this article are solely those of the authors and do not necessarily represent those of their affiliated organizations, or those of the publisher, the editors and the reviewers. Any product that may be evaluated in this article, or claim that may be made by its manufacturer, is not guaranteed or endorsed by the publisher.
