# Supplementary material for: Effect of Radiation Emitted by Wireless Devices on Male Reproductive Hormones: A Systematic Review
Source: Front Physiol. 2021 Sep 24;12:732420. doi: 10.3389/fphys.2021.732420 (PMC8497974; doi:10.3389/fphys.2021.732420)
Supplement: Supplementary file 1 [file Data_Sheet_1.docx]

Supplementary Material

# Supplementary Tables

**Supplementary Table 1** Animal studies - Effect of mobile phone exposure on male reproductive hormones

| Sample size | Animal species/ age | Frequency | Duration of exposure | SAR or Power density | Hormonal findings | | | | References |
| --- | --- | --- | --- | --- | --- | --- | --- | --- | --- |
|  |  |  |  |  | Testosterone | LH | FSH | Others |  |
| N=14;  n=7 | Sprague Dawley rats/ prenatal to 60 days postnatal | 900 MHz EMF generator | 24 h/day for 20 days prenatally until birth | NA | ↓ | NA | NA | NA | Sehitoglu et al. 2015 |
| N=36;  n=12 | Swiss albino mice/3 weeks | 1800 MHz mobile phone | 3 h/day for 120 days | SAR: 0.05 W/kg | ↓ | NA | NA | NA | Shahin et al. 2018 |
| N=16;  n=8 | Wistar rats/30 days | 1835–1850 MHz mobile phone | 1 h/day for 11 weeks | Power density: 1.4 mW/cm^2^ to 0.04 mW/cm^2^ | **↔** | NA | NA | NA | Ribeiro et al. 2007 |
| N=12;  n=6 | Wistar albino rats/70 days | 900 MHz mobile phone | 2 h/day for 45 days | SAR: 0.9 W/kg | ↓ | NA | NA | NA | Kesari & Behari, 2012 |
| N=32;  n=8 | Wistar albino rats/8–10 weeks | 890–915 MHz mobile phone | 12 h/day for 10 weeks | SAR: 0.96 W/kg | **↔** | **↔** | **↔** | **↑** cortisol | Çetkin et al. 2017 |
| N=34;  n=6, 14 | Wistar albino rats/2 months | Mobile phone  (Frequency not mentioned) | 30 or 60 min/day for 3 months | NA | ↓  (in 60 min group) | NA | NA | NA | Meo et al. 2010 |
| N=24;  n=6 | Wistar albino rats/12 weeks | 900 MHz EMF generator | 60 min/day for 28 days | SAR: 2 W/kg  Average power density:1 ± 0.4 mW/cm^2^ | ↓ | NA | NA | NA | Yahyazadeh et al. 2020 |
| N=20;  n=10 | Sprague-Dawley rats/5 months | 900 MHz EMF generator | 30 min/day for 5 days/week for 4 weeks | Average power density: 1 ± 04 mW/cm^2^ | ↓ | **↔** | **↔** | NA | Ozguner et al. 2004 |
| N=20;  n=5 | Sprague–Dawley rats/ 12 weeks | 900 MHz EMF generator | 1, 2, or 4 h/day for 30 days | SAR: 0.19–1.22 W/kg  Average power density: 86 mW/cm^2^ | ↓ | **↑**  **(**in 4 h group) | **↑**  **(**in 4 h group | **↑** prolactin  **(**in 4 h group) | Sepehrimanesh et al. 2013 |
| N=40;  n=10 | C57BL/6 mice/3 months | 900 MHz mobile phone | 8, 16, or 24 h/day for 30 days | NA | ↓  (in 24 h group) | **↑**  **(**in 24 h group) | NA | NA | Zang et al. 2016 |
| N=20;  n-5 | Wistar rats/ not mentioned (initial weight 180–200 g) | Dual-band EGSM 900/1800 MHz mobile phone | 1, 2, or 3 h/day for 28 days | NA | ↓  **(**in 2 and 3 h groups) | ↓  **(**in 2 and 3 h groups) | ↓  **(**in 2 and 3 h groups) | NA | Oyewopo et al. 2017 |
| N=30;  n=10 | Sprague Dawley rat/sexually mature age | 900 MHz radio frequency wave | 2 or 4 h/day for 30 days | SAR: 0.3315 W/kg  Average power density: 0.6789 mW/cm^2^ | ↓ | NA | NA | NA | Azimzadeh & Jelodar 2019 |
| N=18;  n=6 | Purebred New Zealand white rabbits/3 months | 950 MHz, simulated microwave  Output power:3 and 6 W | 2 h/day for 2 weeks | NA | ↓ | NA | NA | NA | Oskouyi et al. 2014 |
| N=21;  N=7 | Wistar albino rats/not mentioned (initial weight 350–400 g) | Mobile phone (Frequency not mentioned) | 4 h/day for 6 weeks | SAR: 0.96 W/kg | ↓ | ↓ | NA | NA | Gevrek et al. 2017 |

NA, not mentioned or conducted in the study; ↓, significant decrease; ↑, significant increase; ↔, no significant difference.

**Supplementary Table 2** Animal studies – Effect of Wi-Fi on male reproductive hormones

| Sample size | Animal species/ age | Frequency | Duration of exposure | SAR or Power density | Hormonal findings | | | | References |
| --- | --- | --- | --- | --- | --- | --- | --- | --- | --- |
|  |  |  |  |  | Testosterone | LH | FSH | Others |  |
| N=15;  n=3 | Wistar albino rats/10 weeks | 2.45 GHz | 2h/day for 60 days | SAR: 0.014 W/kg  Power density: 0.21 mW/cm | ↓ | NA | NA | ↓ melatonin | Kumar et al. 2011 |
| n=48;  n=12 | Sprague-Dawley rats/ not mentioned | 2.45 GHz | 3 h/day for 30 days | SAR: 3.21 W/kg | **↔** | NA | NA | NA | Saygin et al. 2016 |

NA, not mentioned or conducted in the study; ↓, significant decrease; ↑, significant increase; ↔, no significant difference

**Supplementary Table 3** Human studies – Effect of mobile phones on male reproductive hormones

| Sample size | Age of subjects (years) | Frequency | Inclusion/ Exclusion criteria | Study design | Hormonal findings | | | | References |
| --- | --- | --- | --- | --- | --- | --- | --- | --- | --- |
|  |  |  |  |  | Testosterone | LH | FSH | Others |  |
| N=20 | 20–32 | 900 MHz mobile phone  SAR: 0.3 W/kg | Exclusion criteria:  Night-shift or stressful work; frequent exposure to unusually high-intensity EMF at any frequency; mobile phone use prior to entering the study and during the study other than that prescribed by the study; diseases of the ear, nose, and throat; endocrine disorders; neuropsychiatric disease; unusual sleep patterns; and recent transcontinental flight. | Prospective study  Subjects were exposed to RF-EMRs through the use of a mobile phone for 2 h/day, 5 days/week for 4 weeks | **↔** | NA | NA | **↔** cortisol, TSH, growth hormone, prolactin, and ACTH | Djeridane et al. 2008 |
| N=82 | 14–60 | 950 MHz mobile phone | Inclusion criteria:  age 14–60 years, mobile phone users | Prospective study  Group 1 (weak): used mobile phone for less than 10 min/day for 6 years  Group 2 (moderate):  used mobile phone between 30–60 min/day for years  Group 3 (strong): used mobile phone more than 60 min/day for 6 years | ↓ | NA | NA | ↓  ACTH, cortisol, and TSH | Eskandar et al. 2012 |
| N=2110 | Reproductive age;  mean age  31.6 ± 6.6 years | Not mentioned | Inclusion criteria:  Men attending infertility clinic from 1993 to 2007.  Exclusion criteria:  Patients with a history of smoking or alcohol consumption and those with systemic diseases, orchitis, and varicocele. | Retrospective study  Group A: used mobile phone  Group B: did not use mobile phone | **↑** | ↓ | **↔** | **↔** prolactin | Gutschi et al. 2011 |

NA, not mentioned or conducted in the study; ↓, significant decrease; ↑, significant increase; ↔, no significant difference; TSH, Thyroid-stimulating hormone; ACTH, Adrenocorticotropic hormone.

# Supplementary Figure

Records identified through database search

PUBMED (n=56)

MEDLINE (n=395)

Additional records identified through other sources
(n=3)

Identification

Full-text articles assessed for eligibility
(n=21)

Full-text articles excluded, with reasons
(n=2)

- Retracted article (n=2)

Records after duplicate removal
(n=368)

Screening

Records screened
(n=368)

Records excluded
(n=349)

Eligibility

Studies included in qualitative synthesis
(n=19)

Included

**Supplementary Figure 1.** PRISMA flow diagram of the literature search


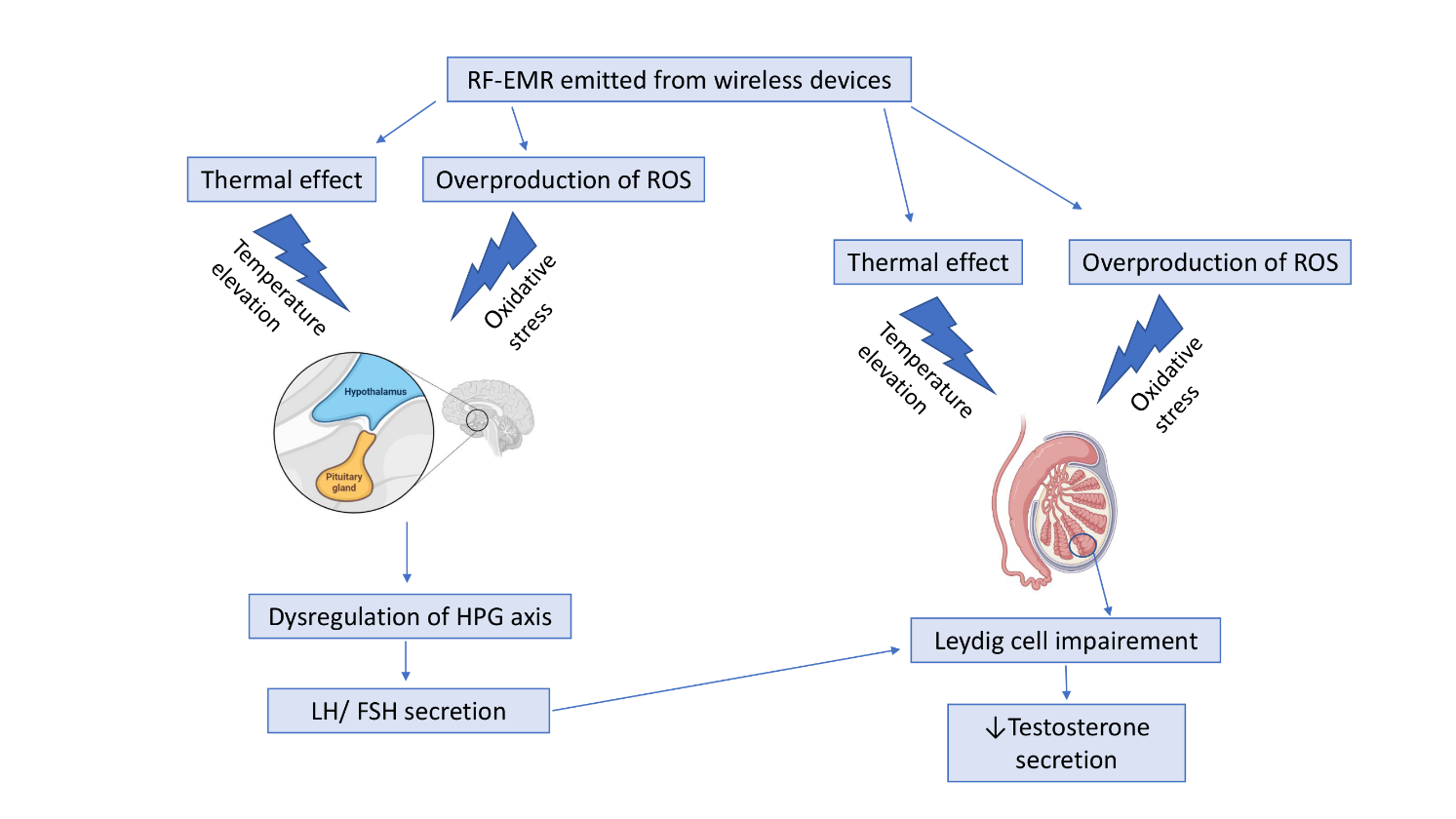


**Supplementary Figure 2.** Potential mechanisms for RF-EMR exposure’s effect on male reproductive hormones. RF-EMR, Radiofrequency electromagnetic radiation; ROS, reactive oxygen species; HPG axis, Hypothalamus-pituitary-gonadal axis; LH, Luteinizing hormone; FSH, Follicle-stimulating hormone.
